# Place of Death From Cancer in US States With vs Without Palliative Care Laws

**DOI:** 10.1001/jamanetworkopen.2023.17247

**Published:** 2023-06-08

**Authors:** Main Lin Quan Vega, Stanford T. Chihuri, Deven Lackraj, Komal Patel Murali, Guohua Li, May Hua

**Affiliations:** 1 Department of Anesthesiology, Columbia University College of Physicians and Surgeons, New York, New York; 2 Department of Physician Assistant Studies, School of Medicine and Health Sciences, George Washington University, Washington, DC; 3 Columbia University School of Nursing, New York, New York; 4 New York University Rory Meyers College of Nursing, New York, New York; 5 Department of Epidemiology, Columbia University Mailman School of Public Health, New York, New York

## Abstract

**Importance:**

In the US, improving end-of-life care has become increasingly urgent. Some states have enacted legislation intended to facilitate palliative care delivery for seriously ill patients, but it is unknown whether these laws have any measurable consequences for patient outcomes.

**Objective:**

To determine whether US state palliative care legislation is associated with place of death from cancer.

**Design, Setting, and Participants:**

This cohort study with a difference-in-differences analysis used information about state legislation combined with death certificate data for 50 US states (from January 1, 2005, to December 31, 2017) for all decedents who had any type of cancer listed as the underlying cause of death. Data analysis for this study occurred between September 1, 2021, and August 31, 2022.

**Exposures:**

Presence of a nonprescriptive (relating to palliative and end-of-life care without prescribing particular clinician actions) or prescriptive (requiring clinicians to offer patients information about care options) palliative care law in the state-year where death occurred.

**Main Outcomes and Measures:**

Multilevel relative risk regression with state modeled as a random effect was used to estimate the likelihood of dying at home or hospice for decedents dying in state-years with a palliative care law compared with decedents dying in state-years without such laws.

**Results:**

This study included 7 547 907 individuals with cancer as the underlying cause of death. Their mean (SD) age was 71 (14) years, and 3 609 146 were women (47.8%). In terms of race and ethnicity, the majority of decedents were White (85.6%) and non-Hispanic (94.1%). During the study period, 553 state-years (85.1%) had no palliative care law, 60 state-years (9.2%) had a nonprescriptive palliative care law, and 37 state-years (5.7%) had a prescriptive palliative care law. A total of 3 780 918 individuals (50.1%) died at home or in hospice. Most decedents (70.8%) died in state-years without a palliative care law, while 15.7% died in state-years with a nonprescriptive law and 13.5% died in state-years with a prescriptive law. Compared with state-years without a palliative care law, the likelihood of dying at home or in hospice was 12% higher for decedents in state-years with a nonprescriptive palliative care law (relative risk, 1.12 [95% CI 1.08-1.16]) and 18% higher for decedents in state-years with a prescriptive palliative care law (relative risk, 1.18 [95% CI, 1.11-1.26]).

**Conclusions and Relevance:**

In this cohort study of decedents from cancer, state palliative care laws were associated with an increased likelihood of dying at home or in hospice. Passage of state palliative care legislation may be an effective policy intervention to increase the number of seriously ill patients who experience their death in such locations.

## Key Points

**Question:**

Is the presence of a state palliative care law associated with dying at home or in hospice among decedents from cancer in the US?

**Findings:**

In this cohort study with a difference-in-differences analysis that included 7 547 907 individuals with cancer as the underlying cause of death, dying in a state-year that had a palliative care law was associated with a 12% to 18% increase in the likelihood of dying at home or in hospice.

**Meaning:**

These findings suggest that state palliative care laws are associated with an increase in the likelihood of dying at home or in hospice among decedents from cancer.

## Teaser

This observational study compares place of death among decedents from cancer in US states with vs without palliative care laws.

## Introduction

In the US, improving palliative and end-of-life care has become an important focus of public health. The size of the older adult population is projected to increase by 80% by 2060,^1^ and both the burden of chronic disease and health care costs are also expected to increase, considering that 25% of total Medicare expenditures are spent during the last year of life.^2,3^ Despite the high cost, care provided at the end of life may not be of high quality or concordant with patient preferences. In the setting of a poor prognosis, the majority of seriously ill patients prefer less aggressive care,^4^ and at the end of life, older adults and their family members value comfort and minimization of suffering, having more time with family, avoiding “being attached to machines and tubes,” and not prolonging death.^5^ Yet many patients die in health care settings,^6^ undergoing high-intensity care that may prioritize quantity over quality of life and prevent timely transition to comfort-focused care, including extensive testing, invasive intervention, and prolonged hospital stays.^7^ This aggressive care may not improve survival,^8^ and it is associated with a decrease in quality of life for patients and worse bereavement adjustment for family members.^9^ Additionally, about 85% of US patients prefer to die at home,^10^ but only 40% die at home or in hospice.^6^ Consequently, place of death is commonly used as an end-of-life care quality metric, under the assumption that dying at home or in hospice is more likely to represent goal-concordant care, and therefore higher-quality care, at the end of life.

One potential solution that has been advocated to improve the discordance between the care that is delivered and the care that most patients believe is optimal at the end of life is the provision of palliative care. Palliative care is defined as “the active, holistic care of individuals across all ages with serious health-related suffering due to severe illness and, especially of those near the end of life. It aims to improve the quality of life of patients, their families, and their caregivers.”^11^ Palliative care encompasses but is not equivalent to end-of-life care, which refers to care delivered to dying patients. Palliative care has been associated with a broad spectrum of benefits, including reductions in caregiver burden,^12^ acute health care utilization and costs, as well as reductions in aggressive care at the end of life.^13^ Additionally, it has been associated with increases in survival and improvements in symptom burden and quality of life for patients with serious illnesses.^13,14,15,16^ For the past 2 decades, there has been a steady increase in the establishment of palliative care in US hospitals.^17^ In further efforts to improve end-of-life care, some states have enacted palliative care legislation aimed at facilitating access to information about available care options as patients approach the end of life, under the assumption that some of the discordant care provided may result from a lack of patient knowledge. However, it is unknown whether these laws have any measurable association with patient outcomes. The aim of this study was to examine the association between the presence of state palliative care legislation and place of death. We hypothesized that in state-years with legislation prescribing clinicians to have discussions with terminally ill patients about their end-of-life care options, decedents would be more likely to die at home or in hospice compared with decedents in state-years without such legislation.

## Methods

### Data Collection and Study Population

This cohort study with a difference-in-differences analysis was deemed by the Columbia University Institutional Review Board as not constituting human participants research and waived the need for informed consent. The study followed the Strengthening the Reporting of Observational Studies in Epidemiology (STROBE) reporting guideline.

Using a natural experimental design,^18^ we analyzed state palliative care legislation data (eTable 1 in Supplement 1) and death certificate data from the US National Center for Health Statistics for January 1, 2005, to December 31, 2017. Death certificate data were obtained for all 50 US states and included a variable denoting the state in which a death occurred. Decedents from the District of Columbia were not included, as the federal government maintains jurisdiction of this city.^19^ The study sample consisted of all decedents in the 50 states who had any type of cancer (based on *International Statistical Classification of Diseases, Tenth Revision*, codes) listed as the underlying cause of death, defined as “the disease or injury which initiated the train of morbid events leading directly or indirectly to death or the circumstances of the accident or violence which produced the fatal injury” (eTable 2 in Supplement 1). We chose to study decedents who died from cancer because use of palliative care has demonstrated positive outcomes in this population and has been formally integrated into oncology guidelines.^20^ We excluded any individuals with an unknown place of death (eFigure in Supplement 1).

The primary outcome of this study was dying at home or in hospice and was determined using place of death from the death certificate data. All other options for place of death (eg, nursing home/long-term care, hospital, clinic or medical center setting, emergency department, dead on arrival, or other) were grouped together. We chose place of death because it is one of the most consistently used quality indicators of end-of-life care, particularly in population-based studies.^21^ Although dying at home or in hospice has been associated with improved quality of death and dying among terminally ill patients with cancer,^22,23,24,25^ this may not be applicable to all patients receiving end-of-life care in all settings.^26^

The primary exposure for this study was the presence of a palliative care law. To determine whether any of the 50 states had legislation related to palliative and end-of-life care during the study period, we performed an online search of publicly available websites that included records of state legislation (largely governmental state public health and state senate websites); the search was completed in September 2021. From these websites, we abstracted whether there was a law on palliative or end-of-life care in place and the date of implementation, a link to the resource, and a brief description of the law. In our search, we identified 2 types of laws: prescriptive and nonprescriptive. Nonprescriptive laws were somewhat related to palliative and end-of-life care but did not prescribe specific clinician actions. For instance, in Georgia, the law established an advisory council: “There is hereby created the Georgia Palliative Care and Quality of Life Advisory Council…. The council shall consult with and advise the department on matters related to the establishment, maintenance, operation, and outcomes evaluation of palliative care initiatives in this state.”^27^

On the other hand, prescriptive laws required clinicians to offer information and counseling to terminally ill patients concerning their care options at the end of life. For example, the New York Palliative Care Information Act states the following:

If a patient is diagnosed with a terminal illness or condition, the patient’s attending health care practitioner shall offer to provide the patient with information and counseling regarding palliative care and end-of-life options appropriate to the patient, including but not limited to: the range of options appropriate to the patient; the prognosis, risks and benefits of the various options; and the patient’s legal rights to comprehensive pain and symptom management at the end of life.^28^

Thus, we created an exposure variable with 3 categories, with decedents being categorized as dying in a state-year with no palliative care law, a state-year with a nonprescriptive palliative care law, or a state-year with a prescriptive palliative care law. We determined exposure status for decedents based on the state and year in which they died.

### Statistical Analysis

We summarized decedent characteristics using descriptive statistics (eg, frequencies and means [SDs]) and calculated standardized differences between groups. Variables that were evaluated for inclusion as potential confounders in adjusted models were decedent age, sex, race, Hispanic ethnicity, education, and marital status. We retrieved race and ethnicity from death certificate data, reported by the funeral director as provided by an informant (often the surviving next of kin), or in the absence of an informant, based on observation.^29^ Race categories were American Indian or Alaska Native, Asian or Pacific Islander, Black, and White. Hispanic ethnicity was categorized into 3 groups: Hispanic, non-Hispanic, or unknown. To examine the association between the presence of palliative care legislation and dying at home or in hospice, we used a multilevel relative risk regression model with state as a random effect. Specifically, the model was estimated using the residual pseudo-likelihood estimation method, a binary distribution and a log link, and state as a random intercept. We chose to include state as a random effect to account for differences in the baseline number of home and hospice deaths in each state; this approach may account for multiple state-level confounders and minimize omitted variable bias. We also adjusted for decedent characteristics with a standardized difference greater than 0.1 between exposure groups. We used 0.1 as a cutoff, as a standardized difference of 10% suggests a negligible correlation (0.05) between treatment group and the binary variable.^30^

We also conducted several secondary analyses. First, we repeated the analysis after omitting data from the year in which a palliative care law was passed. Because the specific date that the law was enacted was often not available and because outcomes resulting from implementation of the law may take time, we omitted these data to decrease the potential of exposure misclassification. Second, we repeated our analysis in a sample of decedents from noncancer serious illnesses, which included patients with chronic obstructive pulmonary disease, end-stage kidney disease, liver disease, heart failure, and dementia as underlying causes of death (eTable 3 in Supplement 1), as these individuals may also benefit from palliative care. Third, we conducted a difference-in-differences analysis. This analysis compares the change in outcomes before and after implementation of legislation in states that enacted a palliative care law to the change in outcomes in states that never had a palliative care law over the same period; this model estimates the association between implementing palliative care legislation and place of death, adjusting for secular trends over time. Because we had 2 exposures, we constructed 2 models. The first model examined the implementation of a nonprescriptive law by comparing the change in place of death in states with nonprescriptive laws (before and after passage of the law) to the change in place of death in states that never implemented any palliative care law. The second model examined the implementation of a prescriptive law by comparing the change in outcome in states with prescriptive laws to that of states that never implemented any such laws; in this analysis, data from state-years with nonprescriptive laws were excluded. Finally, we performed analyses for individual state-years (eTable 4 in Supplement 1) evaluating the association of having a nonprescriptive or prescriptive law (using all states that had no palliative care legislation implemented during the study period as the reference group) with place of death to examine for differences in association strength between state-years.

Statistical significance was set at *P* < .05 for 2-tailed tests. Data management and analysis was conducted using SAS, version 9.4 (SAS Institute Inc), between September 1, 2021, and August 31, 2022.

## Results

The study sample included 7 547 907 individuals with cancer listed as the underlying cause of death. Their overall mean (SD) age was 71 (14) years; 47.8% of decedents were women and 52.2% were men. A total of 0.5% of decedents were American Indian or Alaska Native, 2.6% were Asian or Pacific Islander, 11.3% were Black, and 85.6% were White. In addition, 5.7% of decedents were Hispanic and 94.1% were non-Hispanic (ethnicity was unknown for 0.2%).

 Decedents in state-years with a prescriptive palliative care law were more likely to be of Asian or Pacific Islander race (7.1%), to be of Hispanic ethnicity (13.5%), and to have a marital status of never married or single (10.5%) compared with decedents in state-years with nonprescriptive palliative care laws or no palliative care law. Education level was highly missing in a differential manner (33.4% for decedents in state-years without a palliative care law, 9.7% for decedents in state-years with a nonprescriptive law, and 0% missing for decedents in state-years with a prescriptive law) and therefore was not considered for inclusion in regression analyses (Table). We observed that 553 state-years (85.1%) had no palliative care law, 60 state-years (9.2%) had a nonprescriptive palliative care law, and 37 state-years (5.7%) had a prescriptive palliative care law during the study period (eTable 1 in Supplement 1). Most decedents (70.8%) died in state-years without a palliative care law, while 15.7% died in state-years with a nonprescriptive palliative care law and 13.5% died in state-years with a prescriptive palliative care law.

**Table.** Demographics of Decedents Dying of Cancer, Stratified by Type of Palliative Care Law^a^

Demographic	Palliative care law type		
	None (n = 5 341 279)	Nonprescriptive (n = 1 184 952)	Prescriptive (n = 1 021 676)
Sex			
Standardized difference	NA	−0.02	−0.02
Women	2 540 564 (47.6)	574 417 (48.5)	494 165 (48.4)
Men	2 800 715 (52.4)	610 535 (51.5)	527 511 (51.6)
Age^b^			
Standardized difference	NA	0.01	0.05
Mean (SD)	71 (14)	71 (14)	72 (14)
Race			
Standardized difference	NA	0.25	0.34
American Indian or Alaska Native	30 104 (0.6)	4461 (0.4)	3873 (0.4)
Asian or Pacific Islander	82 865 (1.6)	40 017 (3.4)	72 138 (7.1)
Black	599 171 (11.2)	151 884 (12.8)	102 361 (10.0)
White	4 629 139 (86.7)	988 590 (83.4)	843 304 (82.5)
Ethnicity			
Standardized difference	NA	0.17	0.36
Hispanic	201 614 (3.8)	92 654 (7.8)	137 727 (13.5)
Non-Hispanic	5 130 430 (96.1)	1 089 850 (92.0)	880 126 (86.1)
Unknown	9235 (0.2)	2448 (0.2)	3823 (0.4)
Education			
Grade ≤8, some high school (grades 9-12), or no diploma	729 011 (13.6)	194 111 (16.4)	168 052 (16.4)
High school graduate or completed GED	1 511 928 (28.3)	439 175 (37.1)	382 659 (37.5)
Associate’s or bachelor’s degree or some college credit but no degree	1 046 247 (19.6)	337 838 (28.5)	352 165 (34.5)
Master’s, doctoral, or professional degree	208 568 (3.9)	77 411 (6.5)	88 687 (8.7)
Unknown	62 598 (1.2)	22 076 (1.9)	30 113 (2.9)
Missing	1 782 927 (33.4)	114 341 (9.7)	0
Marital status			
Standardized difference	NA	0.17	0.19
Never married, single	425 250 (8.0)	112 722 (9.5)	107 338 (10.5)
Married	2 685 130 (50.3)	578 131 (48.8)	492 629 (48.2)
Widowed	1 402 919 (26.3)	303 464 (25.6)	250 044 (24.5)
Divorced	809 621 (15.2)	184 183 (15.5)	164 760 (16.1)
Unknown	18 359 (0.3)	6452 (0.5)	6905 (0.7)

Abbreviations: GED, General Educational Development; NA, not applicable.

^a^ Unless indicated otherwise, values are presented as No. (%) of patients.

^b^ Individuals with age not stated or missing (n = 142) were not included in the mean calculation.

For decedents from cancer, 3 780 918 (50.1%) died at home or in hospice. In state-years with prescriptive palliative care laws, 54.3% of decedents died at home or in hospice compared with 50.5% in state-years with nonprescriptive laws and 49.2% in state-years with no palliative care laws. Race, Hispanic ethnicity, and marital status were noted to have standardized differences greater than 0.1 and were included in regression models. After we adjusted for possible confounders, dying in a state and during a year in which either a nonprescriptive (adjusted relative risk [ARR], 1.12 [95% CI, 1.08-1.16]) or prescriptive (ARR, 1.18 [95% CI, 1.11-1.26]) palliative care law was in place was associated with a 12% or 18% increased likelihood of dying at home or in hospice (Figure).

**Figure.**
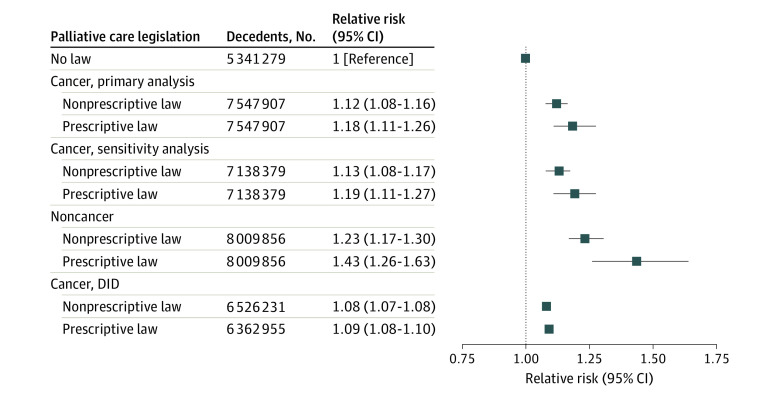
Adjusted Analyses Examining the Association of Palliative Care Legislation With Death at Home or in Hospice Relative risks were adjusted for decedent race, ethnicity, and marital status. State was modeled as a random intercept. The sensitivity analysis excluded decedents from state-years coinciding with implementation of a palliative care legislation. The analysis in decedents with noncancer serious illness included decedents with chronic obstructive pulmonary disease, end-stage kidney disease, liver disease, heart failure, and dementia as underlying causes of death. The difference-in-differences (DID) analysis consisted of 2 separate models to examine state-years before and after implementation of a nonprescriptive palliative care law compared with state-years where no palliative care law was implemented, and state-years before and after implementation of a prescriptive palliative care law compared with state-years where no palliative care law was implemented.

### Secondary Analyses

In an analysis in which we omitted data from the year that palliative care legislation was enacted, results were similar (ARR, 1.13 [95% CI, 1.08-1.17] for nonprescriptive laws and 1.19 [95% CI, 1.11-1.27] for prescriptive laws; Figure). In the secondary sample of decedents from a noncancer serious illness, 27.4% died at home or in hospice. The proportion of decedents dying at home or in hospice was 33.5% in state-years with a prescriptive palliative care law, 28.2% in state-years with nonprescriptive laws, and 26.1% in state-years with no palliative care law. Results of adjusted analyses again demonstrated a positive association between the presence of a palliative care law and dying at home or in hospice, with relative increases of 23% for nonprescriptive laws (ARR, 1.23 [95% CI, 1.17-1.30]) and 43% for prescriptive laws (ARR, 1.43 [95% CI, 1.26-1.63]; Figure). The difference-in-differences analysis yielded similar results, although the magnitude of the association was attenuated (ARR, 1.08 [95% CI, 1.07-1.08] for nonprescriptive laws and 1.09 [95% CI, 1.08-1.10] for prescriptive laws; Figure). Individual state-year analyses showed variability in associations for nonprescriptive and prescriptive laws and place of death (eTable 4 in Supplement 1). For nonprescriptive laws, the association varied between an ARR of 0.79 (95% CI, 0.57-1.11) and 1.33 (95% CI, 0.96-1.86). For prescriptive laws, the association varied between an ARR of 1.07 (95% CI, 0.77-1.49) and 1.38 (95% CI, 0.99-1.92).

## Discussion

In this study examining the association between palliative care legislation and place of death, we observed that dying in state-years with a palliative care law was associated with a small to moderate increase in the likelihood of dying at home or in hospice for decedents from cancer.^31^ These findings were consistent across secondary analyses. We observed a larger association with prescriptive as opposed to nonprescriptive palliative care laws, whereby the relative association was larger for decedents in state-years with prescriptive laws (18%) compared with those in state-years with nonprescriptive laws (12%). In a secondary sample of decedents from noncancer serious illness, we observed a substantial increase in associations between palliative care laws and the likelihood of dying at home or in hospice (relative increases of 23% and 43% for nonprescriptive and prescriptive laws). This apparent increase in association may be due to several factors. Overall, dying at home or in hospice occurred much less frequently for decedents from noncancer serious illness (27.4% vs 50.1% for decedents from cancer), making any absolute change more pronounced on a relative scale. In addition, diagnosis-related disparities in the quality of end-of-life care and lower uptake of palliative care as a standard in this population could translate to a lower baseline of palliative care in decedents from noncancer serious illness,^32,33^ potentially affecting the association of any legislation as an intervention.

In this study, we observed that a state-level intervention had a measurable association with place of death for individual decedents. This finding was noteworthy given the difficulty with improving palliative and end-of-life care delivery in the US.^34^ An effective state-level intervention is appealing, as it necessarily has a broader reach than a patient-level intervention and may require fewer additional resources. Interventions at this level could complement or enhance already established interventions at the individual level, such as the Physicians Orders for Life-Sustaining Treatment or advance care planning, in prompting high-quality communication among clinicians, patients, and their health care proxies to ensure that patients’ goals of care are honored at the end of life.^35,36^

The ostensible mechanism by which a palliative care law might result in an observed change in outcomes is that the law would have its intended outcome of increasing palliative care delivery and conversations about end-of-life care options, resulting in a larger number of people achieving what is assumed to be their goal-concordant choice regarding place of death.^37^ Prior studies have demonstrated that laws or legal proceedings surrounding end-of-life care may potentially affect clinical care. In South Korea, passage of the “well-dying law” was associated with an improvement in the quality of death and dying in South Korean intensive care units (ICUs) as perceived by ICU clinicians.^38^ In Canada, the 2013 Supreme Court decision in *Cuthbertson v Rasouli* deemed the consent of the patient or substitute decision maker mandatory in certain circumstances to withdraw life support.^39,40^ A study using clinical vignettes to compare care choices of Canadian ICU physicians prior to and after the ruling demonstrated that after the *Rasouli* decision, physicians shifted to choosing courses of treatment that were of higher intensity and less “appropriate” (based on their subjective characterization).^39^ However, passage of a law also does not necessarily lead to the desired change in individual behavior. A vignette study examining Australian acute care medical specialists’ care choices in light of laws concerning withholding and withdrawing life-sustaining treatment from adults who lack decisional capacity found that clinician choices were often not legally compliant.^41^ Furthermore, even clinicians who were familiar with the relevant laws stated that their decisions were largely driven by patient factors and not by a desire to be compliant with the law. Together, these studies suggest that while palliative care laws have the potential to affect clinical care and outcomes, the extent is questionable when implemented in an intertwined system where national and institutional culture, institutional policies, clinicians, and patients are all mutually influenced.^42^ Indeed, in individual state-year analyses, we observed heterogeneity with regard to the magnitude of the association between palliative care laws with place of death, suggesting that the extent of outcomes from a palliative care law may be highly dependent on differences in its implementation or environmental factors.

### Limitations

This study has some limitations. The study period did not encompass the most recent data including the latest legislation for states. Furthermore, due to limitations in the data set, we were unable to examine whether passage of palliative care legislation was associated with other markers of palliative care and end-of-life care delivery such as use of palliative care specialists, do-not-resuscitate orders or advance care planning. In addition, we did not have information about individuals’ end-of-life care preferences, information to better understand why laws may or may not have had certain consequences, including information on how legislation may have led to changes in clinical care on a hospital or state level. In choosing dying at home or in hospice as our outcome, we also made an inherent assumption that preferred place of death was home or hospice, which may not be true for all patients. Lastly, there may be residual confounding that was not accounted for by modeling state as a random effect or adjusting for decedent characteristics. In particular, the proportion of deaths occurring in home and hospice has increased over time. However, a difference-in-differences analysis adjusting for this secular trend obtained similar results.

## Conclusions

In this cohort study with a difference-in-differences analysis, dying in state-years with a palliative care law was associated with an increased likelihood of dying at home or in hospice for decedents from serious illnesses. Our findings suggest that palliative care legislation at the state level could be an effective intervention to increase the number of patients with serious illness who experience their death in such locations.

## Notes

Supplement 1. **eTable 1.** Presence and Details of Palliative Care Laws by State, 2005-2017 **eTable 2.** *International Classification of Diseases, Tenth Revision* (*ICD-10*), Diagnosis Codes Used to Identify Decedents With Cancer as the Primary Cause of Death **eTable 3.** *International Classification of Diseases, Tenth Revision* (*ICD-10*), Diagnosis Codes Used to Identify Decedents With Noncancer Serious Illnesses as the Primary Cause of Death **eTable 4.** Estimates of the Association Between Palliative Care Laws and Place of Death for Decedents With Cancer for Individual States **eFigure.** Selection Process of Study Sample

Supplement 2.
Data Sharing Statement
