# Supplementary material for: Place of Death From Cancer in US States With vs Without Palliative Care Laws
Source: JAMA Netw Open. 2023 Jun 8;6(6):e2317247. doi: 10.1001/jamanetworkopen.2023.17247 (PMC10251210; doi:10.1001/jamanetworkopen.2023.17247)
Supplement: Supplement 1. — eTable 1. Presence and Details of Palliative Care Laws by State, 2005-2017 eTable 2. International Classification of Diseases, Tenth Revision (ICD-10), Diagnosis Codes Used to Identify Decedents With Cancer as the Primary Cause of Death eTable 3. International Classification of Diseases, Tenth Revision (ICD-10), Diagnosis Codes Used to Identify Decedents With Noncancer Serious Illnesses as the Primary Cause of Death eTable 4. Estimates of the Association Between Palliative Care Laws and Place of Death for Decedents With Cancer for Individual States eFigure. Selection Process of Study Sample [file jamanetwopen-e2317247-s001.pdf]

Supplementary Online Content

Quan Vega ML, Chihuri ST, Lackraj D, Murali KP, Li G, Hua M. Place of death from cancer in US states with vs without palliative care laws. *JAMA Netw Open*. 2023;6(6):e2317247. doi:10.1001/jamanetworkopen.2023.17247

eTable 1. Presence and Details of Palliative Care Laws by State, 2005-2017

eTable 2. *International Classification of Diseases, Tenth Revision* (*ICD-10*), Diagnosis Codes Used to Identify Decedents With Cancer as the Primary Cause of Death

eTable 3. *International Classification of Diseases, Tenth Revision* (*ICD-10*), Diagnosis Codes Used to Identify Decedents With Noncancer Serious Illnesses as the Primary Cause of Death

eTable 4. Estimates of the Association Between Palliative Care Laws and Place of Death for Decedents With Cancer for Individual States

eFigure. Selection Process of Study Sample

This supplementary material has been provided by the authors to give readers additional information about their work.

© 2023 Vega MLQ et al. *JAMA Network Open*.

eTable 1. Presence and Details of Palliative Care Laws by State, 2005-2017

State	[Law? Y/N]	Date/Year[a]	Link	Description
Alabama	[Y]	May 26, 2015	Palliative_Act.pdf (alabamapublichealth.gov)	State Advisory Council on PC and Quality of Life
Alaska	[N]			
Arizona	[N]			
Arkansas	[N]			
California	[Y]	before 2005	Codes Display Text (ca.gov)	Licensure of hospices by the state department<br>California Hospice Licensure Act of 1990
	[Y]	January 1, 2009	Codes Display Text (ca.gov)	Upon the patient's request, provide the patient with comprehensive information and counseling regarding legal end-of-life care options
Colorado	[N]			
Connecticut	[Y]	October 1, 2013	2013SB-00991-R00-SB.pdf (ct.gov)	Palliative Care Advisory Council within Department of Public Health
Delaware	[N]			
Florida	[Y]	2011	Chapter 765 Section 102 - 2011 Florida Statutes - The Florida Senate (flsenate.gov)	Legislature encourages the professional regulatory boards to adopt appropriate standards and guidelines regarding end-of-life care and pain management and encourages educational institutions established to train health care professionals and allied health professionals to implement curricula to train such professionals to provide end-of-life care, including pain management and palliative care.
	[Y]	2013	Chapter 765 Section 1103 - 2013 Florida Statutes - The Florida Senate (flsenate.gov)	Patient shall be given information concerning pain management and palliative care when he or she discusses with the attending or treating physician,

© 2023 Vega MLQ et al. *JAMA Network Open*.

State	[Law? Y/N]	Date/Year[a]	Link	Description
				or such physician's designee, the diagnosis, planned course of treatment, alternatives, risks, or prognosis for his or her illness.
Georgia	[Y]	July 1, 2016	Section 31-7-192 - Georgia Palliative Care and Quality of Life Advisory Council, Ga. Code § 31-7-192 | Casetext Search + Citator	Palliative Care and Quality of Life Advisory Council
Hawaii	[N]			
Idaho	[N]			
Illinois	[Y]	July 16, 2010	Illinois General Assembly - Illinois Compiled Statutes (ilga.gov)	Hospice and Palliative Care Advisory Board
Indiana	[Y]	2016	Health: Palliative Care Advisory Council (in.gov)	Palliative Care Advisory Council
Iowa	[N]			
Kansas	[N]		Palliative Care & Quality of Life Interdisciplinary Council | KDHE, KS	
Kentucky	[N]			
Louisiana	[N]			
Maine	[Y]	2015	Title 22, §1726: Palliative Care and Quality of Life Interdisciplinary Advisory Council (maine.gov)	Palliative Care and Quality of Life Interdisciplinary Advisory Council
Maryland	[Y]	December 2002	Quality Care at the End of Life, Maryland State Advisory Council	Advisory Council on Quality Care at the End of Life
	[Y]	October 1, 2013	2013 Regular Session - House Bill 581 Chapter (maryland.gov)	Providing for the establishment of a certain number of palliative care pilot programs in certain hospitals in the State
	[Y]	2016	10.07.01.01 and .31 - Proposed Regulation - Department of Health and Mental Hygiene: Hospitals (maryland.gov)	Establish minimum regulatory standards that reflect a consensus on quality practices for palliative care programs within Maryland's hospitals.

© 2023 Vega MLQ et al. *JAMA Network Open*.

State	[Law? Y/N]	Date/Year[a]	Link	Description
	[Y]	March 27, 2017	Palliative Care_10.07.01.31_5_18_2016.pdf (maryland.gov)	Acute general hospitals and special hospitals-chronic care with 50 or more beds shall establish an active hospital wide palliative care program that provides consultation services to patients suffering from pain and symptoms due to serious illnesses or conditions. The hospital or palliative care program shall counsel the palliative care patient or the patient's authorized decision maker regarding specific topics.
Massachusetts	[Y]	January 7, 2015 (Passed)	Session Law - Acts of 2014 Chapter 478 (malegislature.gov)	Palliative care and quality of life interdisciplinary advisory council within the department.
Michigan	[N]			
Minnesota	[Y]	2017	Sec. 57. [144.059] Chapter 6 - MN Laws	Palliative Care Advisory Council
Mississippi	[N]			
Missouri	[Y]	August 28, 2016	Missouri Revisor of Statutes - Revised Statutes of Missouri, RSMo Section 191.1080	Palliative Care and Quality of Life Interdisciplinary Council
Montana	[Y]	2017	50-12-203. Palliative care and quality of life interdisciplinary advisory council -- duties -- membership, MCA (mt.gov)	Palliative care and quality of life interdisciplinary advisory council
Nebraska	[Y]	May 9, 2017 (Approved)	LB323.pdf (nebraskalegislature.gov)	Palliative Care and Quality of Life Advisory Council
Nevada	[Y]	2017	SB136_EN.pdf (state.nv.us)	Advisory Council on Palliative Care and Quality of Life
New Hampshire	[Y]	September 12, 2014	Microsoft Word - sb 0259.doc (state.nh.us)	Establishing a palliative care center for health care consumers and providers
New Jersey	[N]			
New Mexico	[N]			
New York	[Y]<br>[Y]	2007<br>February 9, 2011	Legislation | NY State Senate (nysenate.gov)<br>Bill Search and Legislative Information | New York State Assembly	Palliative care education and training council

© 2023 Vega MLQ et al. *JAMA Network Open*.

State	[Law? Y/N]	Date/Year[a]	Link	Description
		September 27, 2011	Palliative Care Access Act (PHL Section 2997-d) (ny.gov)	If a patient is diagnosed with a terminal illness or condition, the patient's attending health care practitioner shall offer to provide the patient with information and counseling regarding palliative care and end-of-life options appropriate to the patient<br><br>Builds on the Palliative Care Information Act. It applies directly to health care facilities home care agencies, and assisted living residences, as well as individual practitioners. It applies to patients/residents with "advanced life limiting conditions or illnesses who might benefit from palliative care" and not just those who are terminally ill. It requires, not only an offer of information and counseling, but also that the covered health provided or residence "facilitate access to appropriated palliative care consultation and services, including associated pain management consultations and services."
North Carolina	[N]			
North Dakota	[N]			
Ohio	[N]			
Oklahoma	[Y]	April 07, 2015	Bill Information (oklegislature.gov)	The Home Care, Hospice and Palliative Care Advisory Council
Oregon	[Y]	2015	Oregon Health Authority : Palliative Care and Quality of Life Interdisciplinary Advisory Council : Office of Delivery System Innovation : State of Oregon	The Palliative Care and Quality of Life Interdisciplinary Advisory Council
Pennsylvania	[N]			
Rhode Island	[Y]	2013 (Passed)	H5204 (state.ri.us)	Palliative Care and Quality of Life Interdisciplinary Advisory Council
South Carolina	[N]			

© 2023 Vega MLQ et al. *JAMA Network Open*.

State	[Law? Y/N]	Date/Year[a]	Link	Description
South Dakota	[N]			
Tennessee	[N]			
Texas	[Y]	September 1, 2015	84(R) HB 1874 - Enrolled version (texas.gov)	Palliative Care Interdisciplinary Advisory Council
Utah	[N]			
Vermont	[Y]	2009	Vermont Laws	Patient’s Bill of Rights for Palliative Care and Pain Management
Virginia	[N]			
Washington	[N]			
West Virginia	[N]			
Wisconsin	[N]			
Wyoming	[N]			

[a]Date is when law was established/effective unless otherwise specified. In occasions, an exact date was not identified and only the year was available.

© 2023 Vega MLQ et al. *JAMA Network Open*.

eTable 2. *International Classification of Diseases, Tenth Revision* (*ICD-10*), Diagnosis Codes Used to Identify Decedents With Cancer as the Primary Cause of Death

**ICD-10-CM Grouping**	**ICD-10**
Malignant neoplasms of lip, oral cavity and pharynx	C00.X-C14.X
Malignant neoplasms of digestive organs	C15.X-C26.X
Malignant neoplasms of respiratory and intrathoracic organs	C30.X-C39.X
Malignant neoplasms of bone and articular cartilage	C40.X-C41.X
Melanoma and other malignant mesothelial and soft tissue	C43.X-C44.X, C4A.X
Malignant neoplasms of mesothelial and soft tissue	C45.X-C49.XX
Malignant neoplasms of breast	C50.X
Malignant neoplasms of female genital organs	C51.X-C58
Malignant neoplasms of male genital organs	C60.X-C63.X
Malignant neoplasms of urinary tract	C64.X-C68.X
Malignant neoplasms of eye, brain, and other parts of central nervous system	C69.X-C72.X
Malignant neoplasm of thyroid and other endocrine glands	C73.X-C75.X
Malignant neuroendocrine tumors	C7A.X
Secondary neuroendocrine tumors	C7B.X
Malignant neoplasms of ill-defined, other secondary and unspecified sites	C76.X-C80.X
Malignant neoplasms of lymphoid, hematopoietic and related tissue	C81.X-C96.X
In situ neoplasms	D00.X-D09.X
Benign neoplasms, except benign neuroendocrine tumors	D10.X-D36.X
Benign Carcinoid Tumors	D3A.XX
Neoplasms of uncertain behavior, polycythemia vera and myelodysplastic syndromes	D37.X-D48.X
Neoplasm of unspecified behavior	D49.X
Neurofibromatosis, unspecified	Q85.X

© 2023 Vega MLQ et al. *JAMA Network Open*.

eTable 3. *International Classification of Diseases, Tenth Revision* (*ICD-10*), Diagnosis Codes Used to Identify Decedents With Noncancer Serious Illnesses as the Primary Cause of Death

Name of condition	ICD-10
End-stage renal disease	N17-N19
Heart failure	I50.0, I150.1-150.9
Chronic obstructive pulmonary disease	J40, J41-J42, J43, J44
Liver disease	K70, K73, K74
Dementia	F01, F03, G30, R54, I60-I64, I67, I69

© 2023 Vega MLQ et al. *JAMA Network Open*.

eTable 4. Estimates of the Association Between Palliative Care Laws and Place of Death for Decedents With Cancer for Individual States

States[a]	Non-prescriptive law RR (CI)	Prescriptive law RR (CI)
Alabama	1.21 (1.15-1.28)	-
California	[b]	1.10 (0.79-1.54)
Connecticut	0.91 (0.88-0.94)	-
Florida	1.33 (0.96-1.86)	1.38 (0.99-1.92)
Georgia	1.32 (1.29-1.36)	-
Illinois	1.04 (1.00-1.09)	-
Indiana	1.09 (1.06-1.11)	-
Maine	1.20 (1.17-1.23)	-
Maryland	0.84 (0.60-1.17)	1.28 (0.92-1.78)
Massachusetts	1.02 (0.99-1.05)	-
Minnesota	1.02 (0.99-1.04)	-
Missouri	1.11 (1.08-1.14)	-
Montana	1.09 (1.06-1.12)	-
Nebraska	0.87 (0.85-0.89)	-
Nevada	1.29 (1.26-1.32)	-
New Hampshire	1.12 (1.09-1.15)	-
New York	0.79 (0.57-1.11)	[b]
Oklahoma	1.12 (1.09-1.15)	-
Oregon	1.22 (1.18-1.25)	-
Rhode Island	1.21 (1.18-1.25)	-
Texas	1.10 (1.07-1.13)	-
Vermont	-	1.07 (0.77-1.49)

[a] Reference states that have no palliative care law implemented during study years 2005-2017 are not included (Alaska, Arizona, Arkansas, Colorado, Delaware, Hawaii, Idaho, Iowa, Kansas, Kentucky, Louisiana, Michigan, Mississippi, New Jersey, New Mexico, North Carolina, North Dakota, Ohio, Pennsylvania, South Carolina, South Dakota, Tennessee, Utah, Virginia, Washington, West Virginia, Wisconsin, Wyoming). State was modeled as random effect.

[b] Analysis did not converge due to insufficient data points.

© 2023 Vega MLQ et al. *JAMA Network Open*.

eFigure. Selection Process of Study Sample

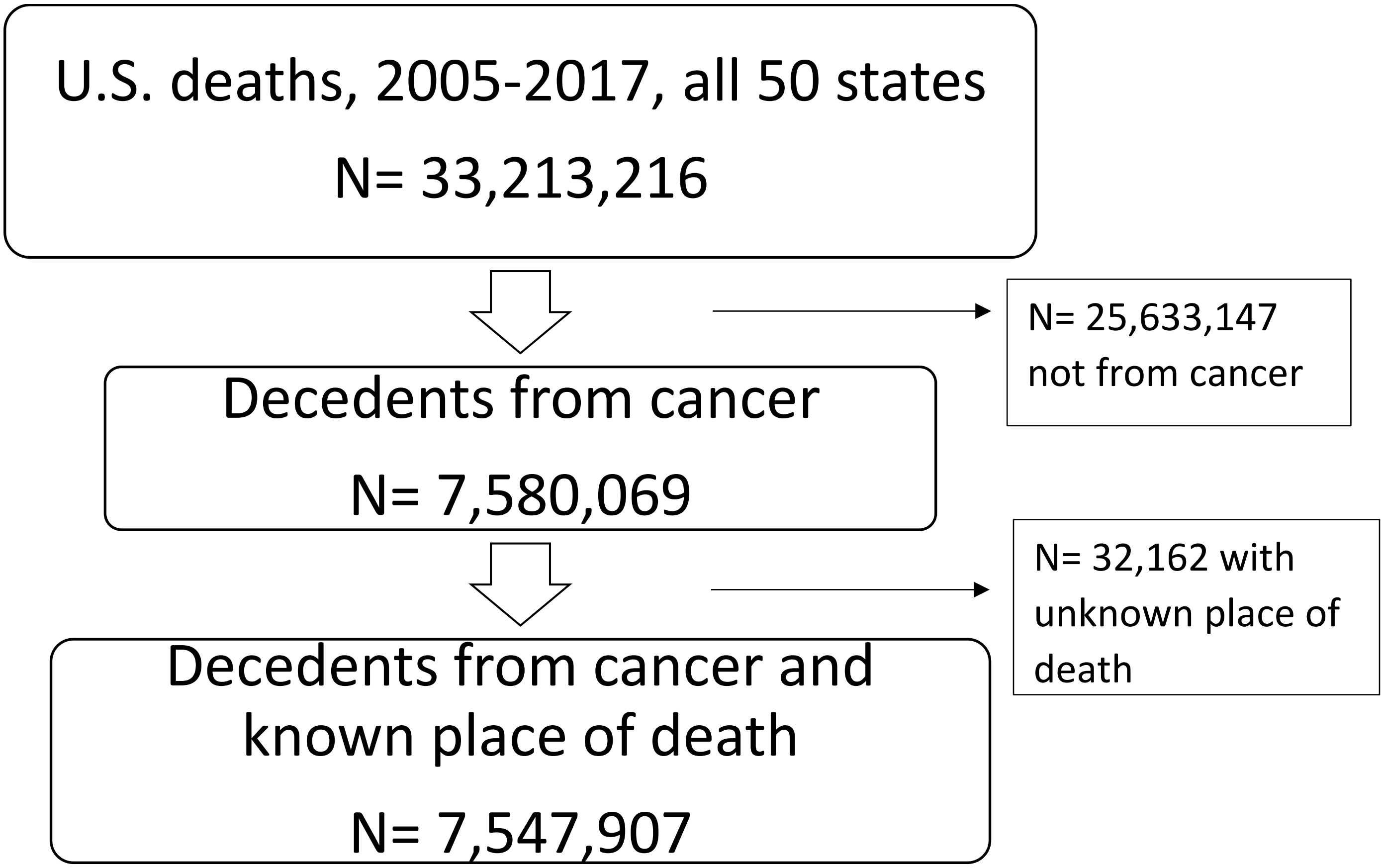

© 2023 Vega MLQ et al. *JAMA Network Open*.
