# Supplementary material for: Place of Death From Cancer in US States With vs Without Palliative Care Laws
Source: JAMA Netw Open. 2023 Jun 8;6(6):e2317247. doi: 10.1001/jamanetworkopen.2023.17247 (PMC10251210; doi:10.1001/jamanetworkopen.2023.17247)
Supplement: Supplement 2. — Data Sharing Statement [file jamanetwopen-e2317247-s002.pdf]

Data Sharing Statement

Quan Vega. Place of Death From Cancer in US States With vs Without Palliative Care Laws. *JAMA Netw Open*. Published June 08, 2023. doi:10.1001/jamanetworkopen.2023.17247

Data
Data available: No

Additional Information
Explanation for why data not available: Data belongs to NCHS
